# An Experimental Study on how Messaging from CDC Affects Attitudes toward Mandatory MMR Vaccination for Schoolchildren

**DOI:** 10.1007/s10900-024-01334-9

**Published:** 2024-02-27

**Authors:** Filip Viskupič, David L. Wiltse

**Affiliations:** School of American and Global Studies, South Dakota State University, Box 2212, Brookings, SD 57007 USA

**Keywords:** MMR vaccine mandate, COVID-19, Survey experiment, Partisan self-identification

## Abstract

**Background:**

During the COVID-19 pandemic, public health institutions, particularly the Centers for Disease Control and Prevention (CDC), were frequently attacked by politicians. Popular trust in these institutions declined, particularly among self-identified Republicans. Therefore, the effectiveness of public health institutions as vaccination messengers might have been weakened in the post-COVID-19 period. We conducted a survey experiment examining the effectiveness of messaging from the CDC in shaping people’s attitudes toward mandatory MMR (measles-mumps-rubella) vaccination for schoolchildren.

**Methods:**

The experiment was embedded in a survey fielded in South Dakota, a “red state” with a population predisposed to distrust the CDC. Using registration-sampling, we received 747 responses. We used difference-in-means tests and multivariate regression to analyze the data.

**Results:**

We found that participants who received a message from the CDC were more likely to support MMR vaccine mandate for schoolchildren than participants who received the same prompt from a state agency. Further analyses showed that messaging from the CDC was particularly effective among Republicans.

**Discussion:**

Overall, our study showed that although the CDC was caught up in the political skirmishes during the COVID-19 pandemic, it remains an authoritative source of public health information.

**Conclusions:**

Public health officials at the local and state levels should not shy away from referring to the CDC in their vaccination messaging.

**Supplementary Information:**

The online version contains supplementary material available at 10.1007/s10900-024-01334-9.

## Introduction

### The Politicization of COVID-19 Mitigation

One of the greatest challenges to the management of the COVID-19 pandemic in the United States was the high degree of politicization [1]. Democrats and Republicans, in the elite and mass electorate alike, offered different assessments of the threat posed by the virus and proposed different solutions. Consequently, there were distinct differences in policy responses between states with Democratic and Republican governors [2, 3]. Similarly, scholars reported sharp differences between Republican and Democratic supporters in adherence to mask-wearing and social distancing [4, 5].

Vaccination was one area of pandemic management that was particularly politicized. Republican Party leaders, particularly President Trump offered only tepid endorsements of COVID-19 vaccination. Studies reported that people who identify as Democrats have greater confidence in COVID-19 vaccines and higher vaccine uptake than self-identified Republicans [6–8].

COVID-19 vaccination mandates likewise became a heated policy question that quickly polarized on partisan terms. Throughout 2021, several states and the federal government imposed mandatory COVID-19 vaccination for certain population groups. Unsurprisingly, Republicans were generally opposed to both targeted vaccination mandates [9, 10], as well as the prospect of a mandate for the general population [11]. The partisan gap in attitudes toward COVID-19 vaccine mandates ran across all sections of society including healthcare workers [12].

## Political Attacks on Public Health Institutions

The political skirmish regarding how to respond to the outbreak of COVID-19 affected public health institutions at the federal, state, and local levels. Politicians, particularly from the Republican Party, were often very critical of these institutions and their policies. Federal-level public health institutions, such as the Centers for Disease Control and Prevention (CDC), bore the brunt of these attacks in the political narrative. For example, President Trump publicly criticized public health guidance regarding the reopening of schools issued by the CDC [13] and called CDC director Dr. Robert Redfield “confused” about vaccines [14]. It was also reported that he used expletives to criticize Dr. Anthony Fauci, then director of the National Institute of Allergy and Infectious Diseases [15]. These attacks persisted for the duration of the public health emergency, and continued after the emergency officially expired. In June 2023, House Republicans accused CDC Director Rochelle Walensky of misrepresenting the effectiveness of COVID-19 vaccines [16]. In many respects, attacks on the CDC and other public health institutions over the COVID-19 response became a staple of Republican politics.

Scholars provided evidence that political attacks during the COVID-19 pandemic undermined people’s trust in public health institutions [17]. A study based on a 2022 survey reported that only a third of the population considered the CDC a highly trusted source of information, while state and local health departments were considered a highly trusted source by about one quarter of the population [18].

The decline in trust was uneven and particularly pronounced among Republicans. Longitudinal studies reported a sharper decline in trust in federal, state, and local governments in the first year of the COVID-19 pandemic among Republicans [19, 20]. Another longitudinal study reported a decline in trust in the CDC among Trump voters during 2020 (Pollard & Davis, 2022). A study based on surveys conducted in New Hampshire found that trust in the CDC fell dramatically among Republicans in the early months of the COVID-19 pandemic [21].

Overall, public health institutions, such as the CDC, were not immune from the political skirmishes during the COVID-19 pandemic. Given the attacks by politicians, trust in these institutions declined, particularly among Republican voters. These institutions, such as the CDC, traditionally played a crucial role in public health messaging, especially regarding vaccination. However, given the developments during the COVID-19 pandemic, their effectiveness as vaccination messengers might be affected in the post-COVID-19 pandemic period, particularly among Republicans.

## This Study

With all this in mind, we explored the effectiveness of the CDC as a messenger regarding vaccination. We conducted a study in South Dakota in the last weeks of the public health emergency examining the effectiveness of messaging from the CDC in shaping people’s attitudes toward mandatory MMR (measles-mumps-rubella) vaccination for schoolchildren. Our study was based on a survey experiment where we test the effects of different messengers on a question of critical public health importance.

Examining attitudes toward an MMR vaccine mandate for schoolchildren can help uncover the extent to which the COVID-19 pandemic affected the role of the CDC as a public health messenger. Scholars have expressed concerns that negative views toward vaccination that people developed during the COVID-19 pandemic might spill over to their attitudes on long-standing vaccines and vaccination mandates [22, 23]. For this reason, it is plausible that the negative views toward the CDC some people developed during the COVID-19 pandemic might have persisted. If that were the case, then messaging from the CDC may negatively affect people’s attitudes toward the measles, mumps, and rubella (MMR) vaccine mandates for schoolchildren – a policy that has enjoyed a great deal of political consensus. In the wake of the first licensing of a measles vaccine in 1963, 17 states had a mandate in place for school enrollment by 1969. By 1980 all states required vaccination for schoolchildren. Additionally, there was broad support for this mandate before the COVID-19 pandemic [24, 25]. If people developed negative attitudes toward the CDC and its effectiveness as public health messenger has been undermined, we would find evidence that invoking the CDC in a message promoting the MMR vaccine would be less effective than a state’s public health authority.

Conducting our study in South Dakota allowed us to get particularly good leverage on our treatment variable. The state’s population is more rural, white, and Republican-leaning compared to the rest of the country. Governor Kristi Noem, a conservative Republican, offered only tepid support for COVID-19 vaccination and was critical of federal public health authorities, such as Dr. Fauci. As such, the population was likely biased against messaging coming from federal-level agencies such as the CDC. South Dakota was therefore a least-likely case in finding evidence for the effectiveness of the CDC as a public health messenger regarding MMR vaccination mandates.

The findings of our study will also be of interest to public health officials, particularly at the local and state levels. They might be considering how to best design communication strategies regarding vaccination, such as MMR vaccination for children, and whether they should include references to the CDC given the backlash that the agency faced during the COVID-19 pandemic. The findings will be particularly relevant for healthcare workers and public health officials based in “red states” or areas within “blue states” with vaccine-hesitant populations.

## Methods

### Data

Our data came from an original online survey. Participants were registered voters in the state of South Dakota. We randomly selected 14,500 people from the list of all registered voters in the state, which we obtained from the Office of the Secretary of the State. Scholars have widely used this method for participant recruitment for cost-effectiveness and representativeness [26]. Those selected were mailed an invitation letter to participate in an online survey hosted on the QuestionPro survey platform. Each letter contained a URL link as well as a QR code to access the survey. Each participant was assigned a unique 6-digit code to open the survey. Data were collected in late March 2023. The response rate was 5.2%, which is on par with similar online surveys using the registration based sampling recruitment method [10, 27]. Participants did not receive any compensation for completing the survey. The authors received approval from the IRB Officer at [University name and approval number redacted for peer review] prior to fielding the survey.

### Experimental Design

Participants were randomly assigned into one of the experimental groups. In one group, participants read a short message about the safety and effectiveness of MMR vaccines for children that was attributed to the CDC. Participants in the second group read the same message, but it was attributed to the South Dakota Department of Health (SDDH). Immediately thereafter, all participants answered the same question about their attitudes toward MMR vaccine mandates for children entering school. We reported the full text of the treatments and all questions in the appendix.

### Measures

We used a single question to measure attitudes toward mandatory MMR vaccination for schoolchildren (1= “strongly oppose” − 5= “strongly support”) that was previously used in other studies [28]. We used a five question scale to measure psychological reactance. The items were taken from the Hong reactance scale [29], and were used by scholars before [30]. The scale included items such as “I am content only when I am acting of my own free will,” which participants answered on a 5-point scale (1 = strongly disagree − 5 = strongly agree). The items were averaged to create one score. The five-item scale showed internal consistency (ω = 0.78) similar to related studies (Albarracin et al., 2021).

We measured partisan self-identification on an ordinal scale of categories valued at 1 (Republican), 2 (independent), and 3 (Democrat). Though partisan self-identification could also be treated as a multinomial variable, it is commonplace in psychology and political science research to measure it ordinally since respondents identifying as “independent” place themselves between the two parties spatially [10, 11, 31].

We also measured age (in years), gender (1 = male, 0 = female and other), education status (six-point ordinal scale from 1 = some high school or less to 6 = post-graduate degree), COVID-19 vaccination status (four-point ordinal scale from 1 = unvaccinated, 2 = one dose of Pfizer or Moderna, 3 = one dose of Johnson & Johnson OR two doses of Pfizer or Moderna, 4 = fully vaccinated and boosted, 5 = Fully vaccinated AND received multiple boosters), feelings thermometers for CDC and SDDH (0-100), and income (1 = under $ 20,000 to 8 = $150,000 or more). The survey also included an attention check, which was passed by 97.3% of participants.

### Analysis

We first offered descriptive statistics of the data. We then conducted two difference-of-means tests to check differences in attitudes toward mandatory MMR vaccination for schoolchildren between the two experimental groups. We then estimated an ordered logistic regression using the same dependent variable and the variables described above served as independent variables. We also calculated predicted probabilities to clarify the effect of the two frames on our dependent variable. We conducted all analyses in Stata [32]. Data weighting was done using the *ebalance* package on age, partisan registration, gender, and region in the state [33]. Post-estimation of ordered logistic analysis was conducted using the *spost* package [34].

## Results

We received 747 responses, of which 52% were male and 48% were female. The average participant age was 56.53 years (SD = 15.85). Over 54% received at least a 4-year college degree and 29% self-identified as evangelical Christians. Regarding political views, 25% of participants identified as Democrats, 31% as Independents, and 44% as Republicans. Over 32% of participants were from the Sioux Falls metropolitan area, and 13% were from the Rapid City metropolitan area. Feelings thermometer score for CDC was 53.76 (SD = 31.53), and the score for SDDH was 61.32 (SD = 20.97). The average score for psychological reactance was 3.20 (SD = 0.83). Over 17% of participants did not receive a COVID-19 vaccination, 2% received only one dose of Pfizer or Moderna vaccines, 15% were fully vaccinated, 20% received a booster dose, and 46% received multiple booster doses.

The overall support for MMR vaccine mandate for schoolchildren was high; 7% of participants were strongly opposed, 4% were somewhat opposed, 8% were neither supportive nor opposed, 15% were somewhat supportive, and 66% were strongly supportive of an MMR vaccine mandate for schoolchildren.

While our data are generally representative of the state population, the sample contained a greater than average number of older adult participants. As we will elaborate in greater detail at the end of the manuscript, we believe that this imbalance is an artifact of the participant recruitment method. For this reason and to account for other possible issues, we used entropy balancing [35] to balance the data on age, gender, partisan self-identification, and region within the state.

Table 1 showed the results of difference-of-means tests in support of MMR vaccine mandate for schoolchildren between participants in the federal and state health agency groups. The results indicated that participants who received a prompt from the CDC were more likely to support MMR vaccine mandate for schoolchildren than participants who receive the same prompt from the SDDH. The difference was statically significant at the 95% confidence interval level (*p* = 0.03, two-tailed test).

**Table 1:** Effect of messenger on support for MMR vaccine mandate for schoolchildren

	Support for MMR vaccine mandate for schoolchildren
Centers for Disease Control	4.29*(4.11, 4.48)
SD Department of Health	4.00 (3.81, 4.19)
Note: 95% confidence intervals in parentheses. ∗ *p* < 0.05 two-tailed test. *N* = 701	

Results of further analyses, shown in Table 2, revealed that messaging from the CDC was particularly effective among some select population groups. Positive and statistically significant effects were observed among Republicans (*p* = 0.001, two-tailed test), adults younger than 65 years (*p* = 0.012, two-tailed test), those who are unvaccinated for COVID-19 (*p* = 0.018, two-tailed test), and those who are fully vaccinated for COVID-19 and already received one booster dose (*p* = 0.018, two-tailed test).

**Table 2:** Effect of messenger on support for MMR vaccine mandate for schoolchildren

	Centers for Disease Control	SD Department of Health
Men (*n* = 370)	4.13 (3.84, 4.41)	3.87 (3.59, 4.14)
Women (*n* = 331)	4.44 (4.19, 4.69)	4.16 (3.90, 4.42)
Democrats (*n* = 167)	4.70 (4.41, 4.98)	4.79 (4.67, 4.92)
Independents (*n* = 206)	4.20 (3.80, 4.61)	3.97 (3.63, 4.32)
Republicans (*n* = 293)	4.24*** (4.00, 4.47)	3.57 (3.26, 3.88)
Younger than 65 years (*n* = 441)	4.25* (4.01, 4.49)	3.81 (3.58, 4.05)
65 years and older (*n* = 260)	4.45 (4.28, 4.62)	4.59 (4.41, 4.74)
COVID unvaccinated (*n* = 121)	3.40* (2.81, 2.99)	2.54 (2.14, 2.94)
COVID fully vaccinated (*n* = 107)	3.63 (3.03, 4.23)	3.71 (3.25, 4.16)
COVID one booster (*n* = 136)	4.73* (4.58, 4.89)	4.35 (4.08, 4.63)
COVID multiple boosters (*n* = 323)	4.80 (4.72, 4.89)	4.83 (4.75, 4.91)
Evangelicals (*n* = 165)	3.59 (3.06, 4.13)	3.40 (3.03, 3.78)
Non-evangelicals (*n* = 425)	4.40 (4.18, 4.61)	4.10 (3.86, 4.34)

Note: 95% confidence intervals in parentheses. ∗ *p* < 0.05, **p < 0.01, ***p < 0.001 two-tailed test

Multivariate analysis results were presented in Table 3. We found a positive and statistically significant relationship between receiving a message from the CDC about MMR vaccines and the attitudes toward MMR vaccine mandate for schoolchildren. The relationships between our dependent variable and age, income, CDC thermometer, partisan self-identification, and COVID-19 vaccination status variables were also positive and statistically significant. The McFadden’s r-square value of 0.211 suggested a well-specified model. The mean variance inflation factor was 1.38 (with the highest individual value of 2.12), indicating no significant levels of multicollinearity.

**Table 3:** Regression results on the effect of CDC messaging on support for MMR vaccine mandate for schoolchildren (*N* = 475)

CDC Prime (0 = SDDH, 1 = CDC)	0.598*
	(0.285)
Age	0.030**
	(0.010)
Male	-0.320
	(0.286)
Education	-0.029
	(0.110)
Evangelical Christian Identity	-0.402
	(0.291)
Income	0.194*
	(0.093)
Partisan Self-Identification	0.235
	(0.216)
CDC Thermometer	0.035***
	(0.006)
Psychological Reactance	-0.097
	(0.191)
COVID Vaccination Status	0.290**
	(0.098)
Number of Cases	475
McFadden’s R-square	0.211
Ordered logistic coefficients with standard errors in parentheses.	
* *p* < 0.05, ** *p* < 0.01, *** *p* < 0.001, two-tailed test.	

Predicted probabilities were estimated for the probability of respondents answering they “strongly support” MMR mandates after they read the statement attributed to one of the two sources. For those that read the statement attributed to the SDDH, the probability of strong support was 0.554. However, for those that read the statement attributed to the CDC, that probability was 0.693, a statistically significant and substantively important difference.

## Discussion

We conducted a fully-randomized survey experiment that investigated the impact of messaging about MMR vaccines from federal and state-level public health institutions on people’s attitudes toward an MMR vaccine mandate for schoolchildren. We found that the participants who received an endorsement from the CDC expressed higher support for the mandate compared to participants who read a message from the SDDH. Further analysis showed that this effect was particularly pronounced among the self-identified Republicans. Our findings make several contributions to the existing literature that should be of interest to both scholars and public health officials.

First, and perhaps foremost, the results showed that even though the CDC has been caught up in the polarization of partisan politics during the COVID-19 pandemic, it remains an authoritative source of public health information. Despite the CDC having a lower mean thermometer score than the SDDH, it proved to be a more effective messenger in our experiment. Our findings speak to the extant research on the credibility of federal versus state agencies in providing public health information. A study based on a nationally representative survey reported that people had greater trust in COVID-19-related information from the state government than from the federal government [36]. Another study found no differences between federal and state public health agencies as trusted sources of information about COVID-19 [37]. Our work suggests that the fears that federal public health agencies may have lost creditability during the pandemic are not always realized. This is particularly true for policy questions such as MMR mandates that have broad bipartisan support. Respondents – even in the heavily Republican state of South Dakota – seem to check their partisanship on this public health policy question and respond positively to a federal messenger. Despite the ideological predisposition of the state’s citizenry towards state over federal authority, our respondents were most moved by a federal messenger.

Second, we found that our Republican respondents proved most responsive to the messaging from the CDC. Given the strong effects of the CDC prime on Republicans, our results suggest that many of the negative predispositions Republicans might have towards the CDC are largely performative. When asked about the CDC itself in our thermometer ratings, they gave relatively low scores relative to the state health department. But, when Republican respondents are asked to contemplate policy preferences beyond highly politicized COVID-19 mitigation, the CDC is still seen as a credible institution. We suspect that the apparent ineffectiveness of the CDC prime on Democrats in our sample was due to ceiling effects. Most Democrats were already strongly supportive of the MMR mandates, leaving little room for improvement irrespective of the prime; whilst independents were simply less likely to follow any partisan cues.

Third, our other findings are in line with the existing literature on the determinants of attitudes toward vaccination. For example, we found that older adults supported MMR vaccine mandate for schoolchildren more than younger adults. Older adults likely have memories of receiving vaccinations at school and were socialized at a time when there was greater consensus on vaccinations in general. Also, older adults are at greater health risks of many diseases that are effectively mitigated by vaccinations. Similarly, those who received a COVID-19 vaccination are also more likely to have positive attitudes toward vaccination and more likely to support mandatory vaccination policy for schoolchildren.

Additionally, the results also showed no impact of partisan self-identification on our dependent variable, suggesting that attitudes toward MMR vaccine mandates are not politicized in the same manner as COVID-19 mitigation practices. Some scholars have expressed concern that the partisan attitudes on COVID-19 vaccines may lead to partisanship structuring attitudes on other vaccine mandates that have historically enjoyed broad consensus (Motta, 2023). While this remains a valid concern our results suggest that this might not be the case for this particular mandate.

Our findings are informative for public health officials at the federal, state, and local levels. Not only should this give some solace to those in the CDC, but it should also indicate to public health officials in other institutions – especially at the state and local level – that they should not shy away from partnering with the CDC in their public health messaging. In fact, given our results, states and localities would be advised not to distance themselves from the CDC in their efforts since their credibility seems to be intact in our rather conservative sample. Future work may want to investigate the potential shallowness of this reflexive and performative opposition to the CDC in the electorate by digging deeper into other policy questions that push respondents away from superficial partisan framing.

### Limitations and Future Directions

We note some limitations of our research. Our survey population comes from South Dakota. The South Dakota population is more ethnically homogenous, rural, and socially and politically conservative than other parts of the United States. While our sample is well suited for our study, it is not nationally representative. Nor is it necessarily representative of all “red” states, since they may not share the same demographics; particularly on ethnic and racial terms.

We also note that while our sample is generally representative of the state population, we were only able to reach those residents of South Dakota who are registered to vote. Therefore, we were not able to reach those residents who are not registered voters or US citizens, or those who just moved and have not updated their voter registration. Table S1 shows that older adults are overrepresented in our sample. This population group is more likely to vote, and this imbalance in our sample is unsurprising and consistent with similar studies (Viskupic & Wiltse, 2022). The greater number of older adults likely accounts for the high support for a MMR vaccine mandate for schoolchildren that we reported. Additionally, our method under-samples the American Indian population of the state given low levels of voter registration. While exploring this question amongst the native population is certainly a worthy enterprise, it is beyond the scope of this study.

We hope that future studies will extend and further develop our findings. As we noted above, while our sample based on the South Dakota population provides a “least likely case” for finding support for the effectiveness of the CDC as public health messenger, similar studies using nationally-representative samples would be particularly welcome.

We suspect that the opposition to the CDC among Republicans might be performative. While self-identified Republicans might voice their disapproval of federal-level agencies such as the CDC either in person or online, they appear to be responsive their advice. While our research design cannot completely explain this phenomenon, we invite scholars to investigate the attitudes this group holds toward public health agencies, particularly at the federal level. We also encourage scholars to examine the effectiveness of federal, state, and local level messengers on other vaccination mandates and health policies.

### Electronic Supplementary Material

Below is the link to the electronic supplementary material.

Supplementary Material 1

## Data Availability

Contact the corresponding author.
