# Supplementary material for: An Experimental Study on how Messaging from CDC Affects Attitudes toward Mandatory MMR Vaccination for Schoolchildren
Source: J Community Health. 2024 Feb 27;49(4):763–9. doi: 10.1007/s10900-024-01334-9 (PMC11306420; doi:10.1007/s10900-024-01334-9)
Supplement: Supplementary file 1 — Supplementary Material 1 [file 10900_2024_1334_MOESM1_ESM.docx]

**Table S1: Weighting Information**

| **Sample Characteristic** | **Unweighted Sample** | **Weighted Sample** |
| --- | --- | --- |
| Male | 0.522 | 0.502 |
| 18 to 24 years | 0.026 | 0.115 |
| 25 to 44 years | 0.218 | 0.332 |
| 45 to 64 years | 0.390 | 0.322 |
| 65 years and over | 0.366 | 0.231 |
| Democrat | 0.250 | 0.255 |
| Republican | 0.443 | 0.416 |
| Sioux Falls metro area | 0.327 | 0.276 |
| Rapid City metro area | 0.131 | 0.129 |

Note: Gender and age information were obtained from census file. Party registration and location information were obtained from the South Dakota voter registration file that the authors obtained from the Secretary of State office.

**Survey Vignettes**

Federal agency group vignette:

"The Centers for Disease Control and Prevention (CDC) recommend that all children between the ages of 1 and 6 receive two doses of the MMR vaccine (measles, mumps, and rubella). Data from the CDC show that the MMR vaccine is safe and highly effective at preventing severe illness and death from these highly infectious diseases."

State agency group vignette:

"The South Dakota Department of Health recommends that all children between the ages of 1 and 6 receive two doses of the MMR vaccine (measles, mumps, and rubella). Data from South Dakota Department of Health show that the MMR vaccine is safe and highly effective at preventing severe illness and death from these highly infectious diseases."

**Survey Questions**

Support for mandatory MMR vaccination for kids to attend school: "Do you support or oppose mandatory MMR vaccination for kids to attend public schools?" (1= "strongly oppose" - 5= "strongly support")

Confidence in MMR vaccine for kids: "How do you think the benefits of the MMR vaccine for kids compare to the risks?" (1= "Risks definitely outweigh the benefits", 2= "Risks likely outweigh the benefits", 3= "Benefits likely outweigh the risks", 4= "Benefits definitely outweigh the risks")

Age: "What was your age on your last birthday?"

Gender: "What is your gender?" (1= male, 2= female, 3=other)

Education: "What is the highest level of education you have completed?” (1= "some high school" - 6= "post-graduate degree")

Income: "What is your total household income in the past 12 months?" (1 = under $20,000, 2 = $20,000 to $39,999, 3 = $40,000 to $74,999, 4 = $75,000 to $99,999, 5 = $100,000 to $149,999, 6 = $150,000 or $249,000, 7 = $250,000 or more, 8 = Prefer not to say)

Evangelical identity: "Would you describe yourself as "born again" or evangelical Christian, or not?" (1= yes, 2=no)

Vaccination status: "What is your COVID-19 vaccination status?" (1= "Fully vaccinated AND received multiple boosters", 2= "Fully vaccinated AND received a single booster", 3= "One dose of Johnson & Johnson OR two doses of Pfizer or Moderna", 4= "Received first dose of Pfizer or Moderna", 5 ="Not vaccinated")

Political party identification: "Generally speaking, do you think of yourself as a Democrat, Republican, an Independent, or something else?" (1= "Democrat", 2= "Republican", 3= "Independent", 4= "Other")

Trust in federal government: "How often can you trust federal government to do what is right?" (1= "Always", 2= "Most of the time", 3= "About half of the time", 4= "Some of the time", 5= "Never")

Trust in state government: "How often can you trust state government to do what is right?" (1= "Always", 2= "Most of the time", 3= "About half of the time", 4= "Some of the time", 5= "Never")

Psychological reactance: "How much do you agree or disagree with the following statements?" (1= strongly disagree - 5= strongly agree)

- It disappoints me to see others submitting to standards and rules.
- I become frustrated when I am unable to make free and independent decisions.
- When someone forces me to do something, I feel like doing the opposite.
- I am content only when I am acting of my own free will.
- It makes me angry when another person is held up as a role model for me to follow.

Attention check: “Please skip this by hitting the "Next" button to move on to the next question. Do not click on the scale below.”
